# Improved *in Vitro* Folding of the Y_2_ G Protein-Coupled Receptor into Bicelles

**DOI:** 10.3389/fmolb.2017.00100

**Published:** 2018-01-17

**Authors:** Peter Schmidt, Brian J. Bender, Anette Kaiser, Khushboo Gulati, Holger A. Scheidt, Heidi E. Hamm, Jens Meiler, Annette G. Beck-Sickinger, Daniel Huster

**Affiliations:** ^1^Faculty of Medicine, Institute for Medical Physics and Biophysics, Leipzig University, Leipzig, Germany; ^2^Center for Structural Biology, Vanderbilt University, Nashville, TN, United States; ^3^Department of Pharmacology, Vanderbilt University Medical Center, Nashville, TN, United States; ^4^Faculty of Life Sciences, Institute of Biochemistry, University of Leipzig, Leipzig, Germany; ^5^Department of Biotechnology, Indian Institute of Technology Roorkee, Uttarakhand, India

**Keywords:** GPCR, NPY, bicelles, folding, NMR

## Abstract

Prerequisite for structural studies on G protein-coupled receptors is the preparation of highly concentrated, stable, and biologically active receptor samples in milligram amounts of protein. Here, we present an improved protocol for *Escherichia coli* expression, functional refolding, and reconstitution into bicelles of the human neuropeptide Y receptor type 2 (Y_2_R) for solution and solid-state NMR experiments. The isotopically labeled receptor is expressed in inclusion bodies and purified using SDS. We studied the details of an improved preparation protocol including the *in vitro* folding of the receptor, e.g., the native disulfide bridge formation, the exchange of the denaturating detergent SDS, and the functional reconstitution into bicelle environments of varying size. Full pharmacological functionality of the Y_2_R preparation was shown by a ligand affinity of 4 nM and G-protein activation. Further, simple NMR experiments are used to test sample quality in high micromolar concentration.

## Introduction

G protein-coupled receptors (GPCRs) play a central role in cell-cell communication and represent the largest group of membrane proteins with over 800 members in the human genome. These molecules transduce signals across the cell membrane via complex formation with extracellular ligands and intracellular interaction partners, namely G-proteins, kinases, and arrestins (Wu et al., 2017). Interaction with intracellular effectors is mediated through structural rearrangements within the seven-transmembrane α-helix bundle and the loops connecting these α-helices. The dynamic nature of these binding processes has recently been shown in structural detail for the ß2-adrenergic receptor (Manglik et al., 2015) and the A2A adenosine receptor (Ye et al., 2016). Influencing these signal transduction pathways holds great potential for pharmaceutical research. Active components in several of the highest selling FDA approved pharmaceutical products in 2016 directly act on GPCRs, for instance in the treatment of depression, asthma, or pain (see fda.gov). Structure based design of highly specific agonists and antagonists targeting GPCRs with reduced side effects requires comprehensive knowledge about the structure and dynamics of these membrane embedded molecules at different stages in their signaling process.

To date, over 150 crystal structures from 35 individual GPCRs in different activation states have been deposited in the protein database providing a large body of available data regarding structural features of GPCRs as recently reviewed (Wu et al., 2017). In spite of the significant breakthroughs these crystal structures provide for the GPCR field, they represent static views, typically achieved in a non-native environment. Furthermore, for crystallography, GPCRs are typically engineered to stabilize one conformation and/or aid crystallization. Alterations include truncation of flexible regions, extensive mutagenesis (Warne et al., 2008; Egloff et al., 2014), introduction of additional disulfide bonds (Standfuss et al., 2011), or replacement of loops with stabilizing proteins (Rosenbaum et al., 2007). These static and artificially stabilized snapshots of GPCRs can only partially reveal the rich dynamical features of these molecules. Therefore, non-crystallographic biophysical tools are required to fully characterize the dynamics of these flexible and conformationally complex membrane proteins (Kobilka and Schertler, 2008; Latorraca et al., 2017).

Complementary to standard crystallography and, more recently, cryo-electron microscopy (Liang et al., 2017; Zhang et al., 2017), NMR spectroscopy represents a versatile method to obtain structural information on both non-engineered GPCRs in a membrane (mimicking) environment (Warschawski et al., 2011) and also of their ligands in complex with the receptors (Lopez et al., 2008; Catoire et al., 2010; Kaiser et al., 2015). Both, solution and solid-state NMR spectroscopy provide complementary NMR constraints for GPCR research (Isogai et al., 2016; Ye et al., 2016). For example, chemical shift perturbation (CSP) measurements using solution NMR provided information about binding events in the receptor/water interface on G-protein in complex with the neurotensin-1 receptor (Goricanec et al., 2016). In solid-state MAS NMR, the strengths of dipolar couplings were measured to obtain through space distance information for determining a structural model of the CXCR1 receptor (Park et al., 2012b) or characterizing the Y_2_R dynamics (Schmidt et al., 2014). Furthermore, the complementary use of restraints from solution and solid-state MAS NMR was demonstrated for the structure modeling of neuropeptide Y (NPY) in complex with its Y_2_R (Kaiser et al., 2015).

In all the NMR studies mentioned above, the GPCRs were obtained from prokaryotic expression in *Escherichia coli*. Either the receptors were expressed functionally (Vukoti et al., 2012), stabilized by directed evolution (Schlinkmann and Pluckthun, 2013), or non-functionally in inclusion bodies (Schmidt et al., 2009; Park et al., 2012a). The latter method provides a feasible and economical method to express the required milligram amounts of non-engineered, isotopically labeled GPCRs for NMR studies. However, the molecules aggregated in inclusion bodies must subsequently be solubilized and folded *in vitro* into their functional state (Baneres et al., 2011). A number of studies using refolding of GPCRs into lipid environment have been published and demonstrated that valuable information on structure (Park et al., 2012b) or dynamics (Schmidt et al., 2014; Schrottke et al., 2017) can be obtained for the receptors alone or in complex with intracellular (Damian et al., 2015) or extracellular (Kaiser et al., 2015) binding partners. Nevertheless, developing efficient and successful folding protocols remains challenging and time consuming as each individual step in the refolding protocol introduces obstacles that must be overcome by optimization.

Of course, the benchmark indicating the success of the refolding protocol of GPCR samples for NMR studies are functionality assays of the folded molecules at various concentrations. In most studies, functionality of the GPCRs, irrespective if refolded or functionally expressed, is measured in radioligand binding assays. These assays are performed at nanomolar receptor concentrations in order to determine the low nanomolar ligand affinities and to avoid the extensive use of expensive radioactive labeled material. However, in NMR measurements, GPCR concentrations in the high micro- to low millimolar range have to be used. During the necessary procedures to increase receptor concentration, the stabilizing environmental properties likely change with respect to protein/lipid or protein/detergent ratios, total receptor concentration, or solvent viscosity and might denature the protein and/or lead to protein aggregation. Hence, functionality should be confirmed at the protein concentrations required for structural measurements using the respective method.

Here, we present in detail an optimized three-step folding protocol of the human neuropeptide Y type 2 receptor (Y_2_R) into phospholipid bicelles, providing samples for both solution and solid-state MAS NMR experiments. The Y_2_R is involved in the regulation of a number of physiological processes including food intake, neuroprotection, and circadian rhythm. As a consequence, the Y_2_R is a putative target for therapeutics to treat obesity, epilepsy, schizophrenia, or anti-social behavior like aggression, depression, and drug addiction (Parker and Balasubramaniam, 2008). Furthermore, we show binding of the ligand neuropeptide Y (NPY) to the Y_2_R as well as competence of the activated Y_2_R to catalyze nucleotide exchange in Gi-proteins using concentrations from the high nano- to the micromolar range using fluorescence and NMR spectroscopy.

## Materials and methods

### Y_2_R sample preparation

Expression of a cysteine deficient variant of the human Y_2_R (Witte et al., 2013) in *E. coli* as inclusion bodies, receptor solubilization, and IMAC purification in 15 mM sodium dodecyl sulfate (SDS), 50 mM sodium phosphate (NaP), yielding ~20 mg Y_2_R per liter of expression medium, were performed as described before (Schmidt et al., 2010).

**Figure 1:**
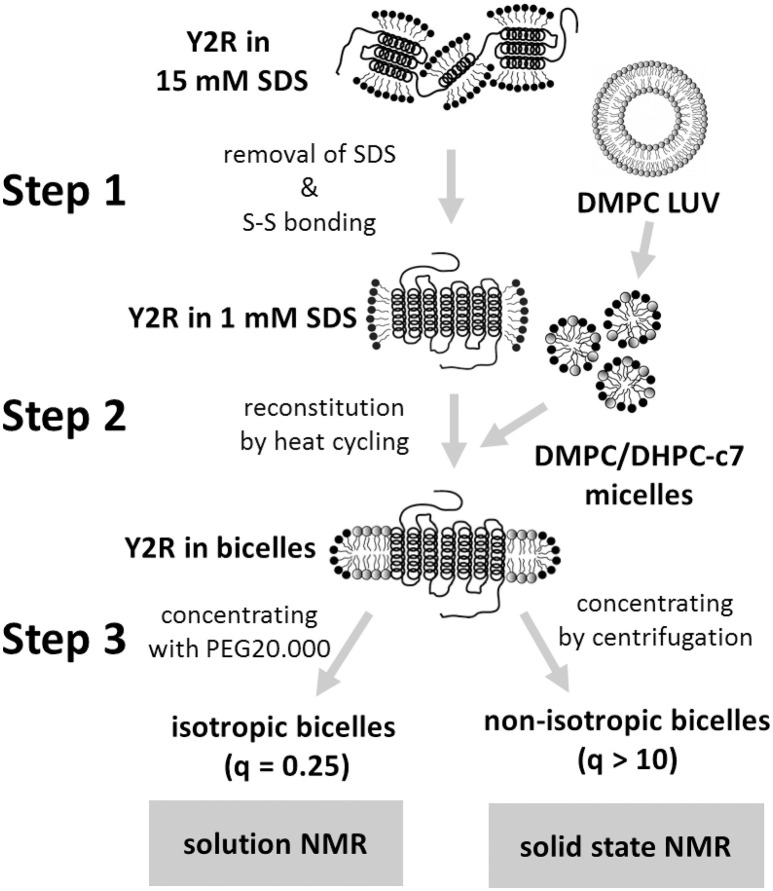
Scheme of the three-step folding protocol for the preparation of the Y_2_R in either isotropic or non-isotropic bicelles. The main steps are the folding dialysis (step 1), the reconstitution into bicelles (step 2), and concentrating the sample for NMR measurements (step 3).

To refold the Y_2_R into a functional state, a three-step folding protocol was developed (Figure 1), which is explained in detail in the results section. The following buffers were used: in step 1, the purified Y_2_R is dialyzed against a carefully degassed buffer containing 1 mM SDS, 50 mM NaP at pH 8.5, 1 mM EDTA, 1 mM reduced glutathione (GSH), and 0.5 mM oxidized glutathione (GSSG) at room temperature for 48 h using dialysis tubing with an 8–10 kDa molecular weight cut-off. Subsequently, 25 wt% poly(ethylene glycol) of a molecular weight of 20 kDa (PEG 20,000) is added to the same buffer to concentrate the receptor before reconstitution. In step 2, preformed bicelles consisting of 1,2-dimyristoyl-*sn*-glycero-3-phosphocholine (DMPC) and 1,2-diheptanoyl-*sn*-glycero-3-phosphocholine (DHPC-c7) (obtained from Avanti Polar Lipids, Alabaster, USA) at a DMPC/DHPC-c7 molar ratio of 1:4 (*q*-value of 0.25) and dissolved in 50 mM NaP at pH 8.0 were incubated with the Y_2_R, followed by three cycles of fast temperature changes from 42 to 0°C with an incubation time of 25 min each. Visibly aggregated protein at any stage of the refolding protocol was removed instantly by centrifugation. In step 3, the Y_2_R samples were either concentrated in small (*q* = 0.25) or large bicelles (*q* > 10). For small bicelle preparations, the samples were dialyzed at least three times against solutions containing 20–30 wt% PEG 20,000, 1.5 mM DHPC-c7, 50 mM NaP at pH 7. For large bicelle preparations, 50 mg/ml BioBeadsSM2 were added at least twice to the solution until the sample became slightly turbid. After removal of the beads with a sieve, the samples were washed four times through cycles of pelleting by centrifugation and resolubilization in 50 mM NaP at pH 7. Concentration determination of the membrane embedded receptors was performed by solubilization of the bicelles in 10 fold volume of 15 mM SDS, 50 mM NaP at pH7 and subsequent measurement of the Y_2_R intrinsic absorption at 280 nm using UV-Vis.

### Negative stain electron microscopy

Y_2_R prepared in bicelles of varying *q*-values were diluted to 0.3–0.5 μM in 50 mM NaP, pH 7, 1 mM EDTA and 1.5 mM DHPC (only for low *q*-values). A 3 μL sample was adsorbed onto a glow-discharged copper grid coated with a carbon film. The samples were washed with two drops of water and stained in two drops of uranyl formate (0.75%). Samples were visualized on a FEI Morgani electron microscope equipped with a 1 × 1 k ATM CCD camera. The electron dose was set to 100 kV and magnification to 28,000×, unless otherwise noted.

### Assessment of disulfide bridge formation

To monitor the disulfide bridge formation, free cysteines were labeled with thiol-specific fluorochrome N-[4-(7-diethylamino-4-methyl-3-coumarinyl)phenyl]maleimide (CPM) (Alexandrov et al., 2008). A stock solution of CPM was dissolved in DMSO (4 mg/mL). The solution used in the experiments was further diluted using a 40 fold excess of buffer. A total of 10 μg of Y_2_R collected at various time points were diluted in buffer containing 15 mM SDS to a final volume of 720 μL. A volume of 60 μL of the working stock solution of CPM was added to the Y_2_R, and incubated at room temperature in the dark for 15 min. Data were collected on FluoroMax-2 (JOBIN YVON) in a 10 mm quartz cuvette at 20°C with an excitation wavelength of 387 nm, scanning emission wavelength from 450 to 500 nm, and integration time 0.5 s. All samples were scanned three times.

### Fluorescence polarization ligand binding assay

Functionality of the Y_2_R in nanomolar concentration was verified in a fluorescence polarization binding assay (Casiraghi et al., 2016; Schrottke et al., 2017) using [Dpr^22^-atto520]-NPY. The reconstituted Y2 receptor was incubated in increasing concentrations with the fluorescently labeled NPY at a concentration of 50 nM overnight at room temperature in 50 mM NaP at pH 7 in duplicate. The fluorescence spectra were recorded on the FluoroMax-2 using a 10 mm quartz cuvette at 20°C. The polarization units for each point were calculated from the maximal intensities of the four spectra measured in different planes and plotted against the receptor concentration as described in the literature (Lea and Simeonov, 2011). As control, NPY binding to empty bicelles in the same concentrations as the receptor-containing bicelles were measured. In competition assays, constant concentrations of 50 nM Y_2_R, 50 nM attoNPY and increasing concentrations of unlabeled NPY were used.

### G-protein activation *in vitro*

Wild type Gα_i1_ protein was produced in *E. coli* and purified as described in the literature (Medkova et al., 2002; Alexander et al., 2014). Protein was stored at a concentration of 50 mM in Tris-Cl buffer, pH 8.0, 50 mM NaCl, 2 mM MgCl_2_, 1 mM dithiothreitol, 10 μM guanosine diphosphate (GDP), and 10% glycerol at −80°C. Gβ_1_γ_1_ protein was isolated from bovine rod outer segments as described earlier (Mazzoni et al., 1991) and stored at a concentration of 10 mM in Tris-Cl buffer, pH 7.5, 100 mM NaCl, 5 mM 2-mercaptoethanol, and 10% glycerol at −80°C.

Nucleotide exchange in the basal state (Gα only) or catalyzed by activated receptor (R^*^-Gαβγ) was monitored as increase of intrinsic tryptophan fluorescence of W^211^ within switch II of Gα_i_ (Hamm et al., 2009) following binding to non-hydrolyzable GTPγS. Measurements were carried out at 16°C in semi-micro cuvettes (109.004F, Hellma, Müllheim, Germany) under constant magnetic stirring in a LS 50B fluorescence spectrometer (Perkin Elmer, Waltham, MA, USA), kinetic mode, constant photomultiplier voltage of 750 V, using excitation and emission filters of λ_ex_ 290/5 nm and λ_em_345/5 nm, signal integration time of 800 ms, and signal interval of 2 s. For measurement of basal nucleotide exchange, fluorescence increase of 200 nM Gα (0.24 nmol in 1200 μl total volume) in 50 mM NaP/DHPC degassed buffer was monitored after addition of 82 μM GTPγS (10 μl of 10 mM stock in H_2_O). For measurement of receptor-catalyzed nucleotide exchange, Gαβ_1_γ_1_ (0.24 nmol; 10% molar excess of β_1_γ_1_) was pre-assembled in 10 μl Tris-Cl pH 7.5, 50 mM NaCl, 1 mM MgCl_2_ for 10 min on ice. Y_2_R (0.24 nmol) was activated with 10 fold excess of NPY in 20 μl NaP/DHPC (NaP for non-isotropic Y_2_R preparations) for 30 min at room temperature. Y_2_R-NPY was allowed to bind pre-formed Gαβ_1_γ_1_ for 10 min at 15°C, and the complex was added to the cuvette preloaded with degassed NaP/DHPC (NaP for non-isotropic Y_2_R preparations). Samples were equilibrated in the cuvette for 5 min to ensure a stable baseline, and 82 μM GTPγS was added. GTPγS binding kinetics was fitted applying the built-in one-phase association function of GraphPad Prism 5.03 (GraphPad Software, San Diego, CA, USA) to obtain the apparent rate constant k.

### Peptide synthesis

Porcine NPY and isotopically labeled NPY variants were synthesized by combined manual/automated fluorenylmethyloxy-carbonyl/*tert*-butyl (Fmoc/*t*Bu) solid phase peptide synthesis in 15 μM scale on Rink amide resin as described before (Beck-Sickinger et al., 1994). Fluorescently labeled NPY [Dpr22-atto520]NPY was synthesized as decribed (Schrottke et al., 2017). Peptides were purified on a preparative reversed-phase high-performance liquid chromatography (RP-HPLC) system with C18 column (Jupiter 10U Proteo, Phenomenex, Aschaffenburg, Germany), applying linear gradients of 0.1% TFA in H_2_O (eluent A) and 0.08% TFA in ACN (eluent B).

### NMR measurements

All NMR spectra were acquired on a on a Bruker 600 Avance III NMR spectrometer (Bruker BioSpin GmbH, Rheinstetten, Germany) at a resonance frequency of 600.1 MHz for ^1^H, 150.9 MHz for ^13^C, and 60.8 MHz for ^15^N. Solution state experiments were conducted using a standard TXI probe. For solid-state NMR measurements, either a 4 mm MAS double or a 3.2 mm MAS triple resonance probe was used. Typical 90° pulse lengths for both probes were 4 μs for ^1^H and ^13^C and 5 μs for ^15^N. ^1^H dipolar decoupling during acquisition with and radio frequency amplitude of 65 kHz was applied using Spinal64. Chemical shifts were referenced externally (for ^13^C relative to TMS). For the ^13^C-^13^C DARR spectra, a CP contact time of 700 μs and a mixing time of up to 500 ms was used. In the indirect dimension, 180 increments were accumulated. The relaxation delay was 2 s.

## Results

### *In vitro* folding of the Y_2_R into bicelles

After *E. coli* expression and solubilization in SDS for IMAC purification, the Y_2_R is ready for refolding. Figure 1 shows a scheme of the three-step folding process for the Y_2_R to remove the denaturating SDS, form the native disulfide bridge, reconstitute into stable lipid environment, and finally to obtain concentrated samples of functional Y_2_R in either isotropic bicelles (*q* = 0.25) for solution NMR or non-isotropic bicelles (*q* > 10) for solid-state MAS NMR studies. The folding process comprises three main steps; step 1—the folding dialysis, step 2—the reconstitution into bicelles, and step 3—concentrating the sample for NMR in either isotropic or non-isotropic bicelles.

In step 1, the SDS concentration is reduced to just below its critical micelle concentration (CMC) by dialysis. This reduction of the SDS/receptor molar ratio enables the formation of intramolecular contacts between the receptor α-helices and allows a pre-formation of the α-helical bundle. Best results, characterized by less than 10% protein aggregation, were achieved when using a concentration of 10 μM for the Y_2_R and 1 mM for the SDS in the dialysis resulting in a SDS/Y_2_R molar ratio of 100. Using lower SDS/Y_2_R ratios resulted in lower reconstitution yields in the subsequent folding step 2, while higher ratios lowered the proportion of active protein. Additionally, the glutathione based redox-shuffling system was added at this step to ensure the formation of the native disulfide bridge in this cysteine reduced variant of the Y_2_R (Witte et al., 2013).

Prior to reconstitution in step 2, the Y_2_R is concentrated to 20–30 μM by adding PEG 20,000 to the dialysis buffer. Through this concentration step, the sample volume as well as the SDS/Y_2_R ratio is reduced, which slightly improved reconstitution yields. More importantly, the reduction of the sample volume simplifies and accelerates the concentration in step 3 when preparing samples for solution NMR measurements. At low SDS concentration, the concentrated receptor molecules are more prone to oligomerize and the step 2 reconstitution has to be performed directly afterwards. Reconstitution is achieved by addition of freshly prepared DHPC-c7/DMPC mixed micelles, solubilized to a *q*-value of 0.25 from preformed DMPC vesicles of 100 nm diameter and a concentration of 10 mg/ml DMPC (Schmidt et al., 2010). The DMPC/Y_2_R molar ratio depends on the final desired preparation. For the preparation of non-isotropic bicelles, a ratio of 180/1 is used, while for isotropic bicelles, a ratio of 400/1 shows the best results. Ratios below these values drastically reduce either the reconstitution yield or, in case of the isotropic bicelles, the stability in the final sample after concentrating the sample in step 3.

Step 2, reconstitution of the Y_2_R from the low SDS concentration environment into the DMPC bilayer, is achieved through a heat cycling process (De Angelis and Opella, 2007), where the solution containing the receptors in SDS micelles and the DMPC/DHPC-c7 bicelles is alternately heated and cooled well above and below the phase transition of the lipid/detergent mixture, respectively. This procedure alters the lateral forces acting on the receptor between mixed micelles and isotropic bicelles or rather between bilayer and non-bilayer formation, and hence facilitates the replacement of the high cmc SDS detergents by the very low cmc DMPC lipids on the hydrophobic core of the receptors, which should form a stable bilayer around the α-helical receptor bundle. The unfavorable SDS is replaced by a zwitterionic phospholipid bilayer applying a well-defined lateral pressure profile onto the receptor (Marsh, 1996) and finally allows for the native orientation of the transmembrane helices in the membrane mediated by side chain contacts with lipids. At the end of step 2, the Y_2_R is stabilized in the isotropic bicelle solution at a concentration of 15–20 μM.

In step 3, the sample has to be concentrated to the micromolar range required for NMR measurements, and impurities such as residual SDS molecules, glutathione from redox-shuffling system, and EDTA, which disturb the NMR measurements, have to be removed. The procedure for sample concentration depends on the measurements the sample is prepared for. For solution NMR experiments, it is important to maintain the isotropic bicelles at a *q*-value of 0.25. Therefore, the Y_2_R sample is concentrated by dialysis against a buffer containing PEG 20,000 for the removal of the water and DHPC-c7 slightly above the CMC for maintaining the receptor/lipid/detergent ratio. Multiple buffer exchanges assure the removal of SDS, glutathione, and EDTA. A sample stability of at least 48 h, which is required for the solution NMR spectra shown below, was achieved with samples concentrated up to 160 μM Y_2_R. At longer time periods or higher concentration, the isotropic bicelles started to fuse or aggregate to larger lipid complexes, and were not useful for solution NMR measurements indicated by substantial line broadening.

**Figure 2:**
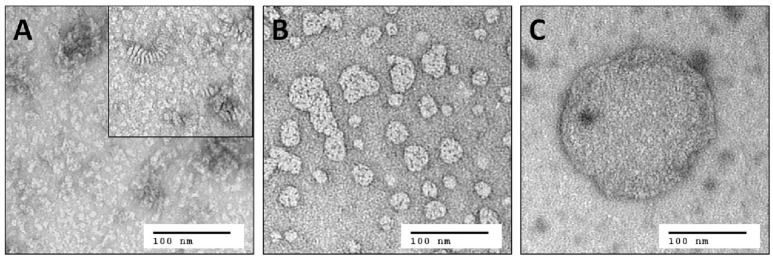
Negative staining electron microscopy images of **(A)** small isotropic bicelles (*q* = 0.25), **(B)** intermediate sized bicelles, and **(C)** non-isotropic bicelles (*q* > 10). The inset in **(A)** shows the same sample after 1 week of storage at room temperature. Stacking of the bicelles becomes visible, which leads to reduced binding yields and substantial line broadening in solution NMR spectra. Samples from **(A)** and **(C)** are used for solution and solid-state MAS NMR, respectively. Image **(B)** illustrates the fusion of the small bicelles to larger patches during removal of the DHPC-c7 and hence to an increased *q*-value.

In contrast to solution NMR, large complexes are well-suited for solid-state MAS NMR measurements. Here, one limitation is the total amount of receptor in the sample, and hence the signal intensity, which is constrained by the available volume of the MAS rotor. The concentration of active protein can consequently only be increased by reducing the amount of all other components in the sample such as lipid and water. As stated above, the lowest DMPC/Y_2_R ratio, required for functional reconstitution in step 2, was 180/1. The DHPC-c7 on the other hand, which is required for reconstitution in step 2 and for maintaining isotropic bicelles conditions for solution NMR measurements in step 3, is not necessary for the functionality of the Y_2_R once embedded in the bilayer. Therefore, the detergent is removed in multiple incubation steps using BioBeadsSM2 (Rigaud et al., 1997), which changes the *q*-value from 0.25 to above 10 and facilitates the fusion of the bicelles from small structures to large, non-isotropic bicelle-like patches with a diameter of 300–500 nm, as visualized in Figure 2. The DMPC/DHPC-c7 ratio was determined from one-dimensional ^1^H solid-state MAS NMR spectra. The large non-isotropic bicelles can easily be pelleted by centrifugation, which is used for the removal of SDS, glutathione, and EDTA using several washing steps, and for concentrating the sample. The final Y_2_R sample for solid-state NMR measurements contained ~6 mg Y_2_R in a 50 μl NMR rotor with a water content of ~50%, determined by weighing before and after lyophilization. In contrast to the isotropic bicelle samples, the Y_2_R embedded in non-isotropic bicelles was stable for at least 1 month at −20°C, displaying no changes in the NMR spectra.

**Figure 3:**
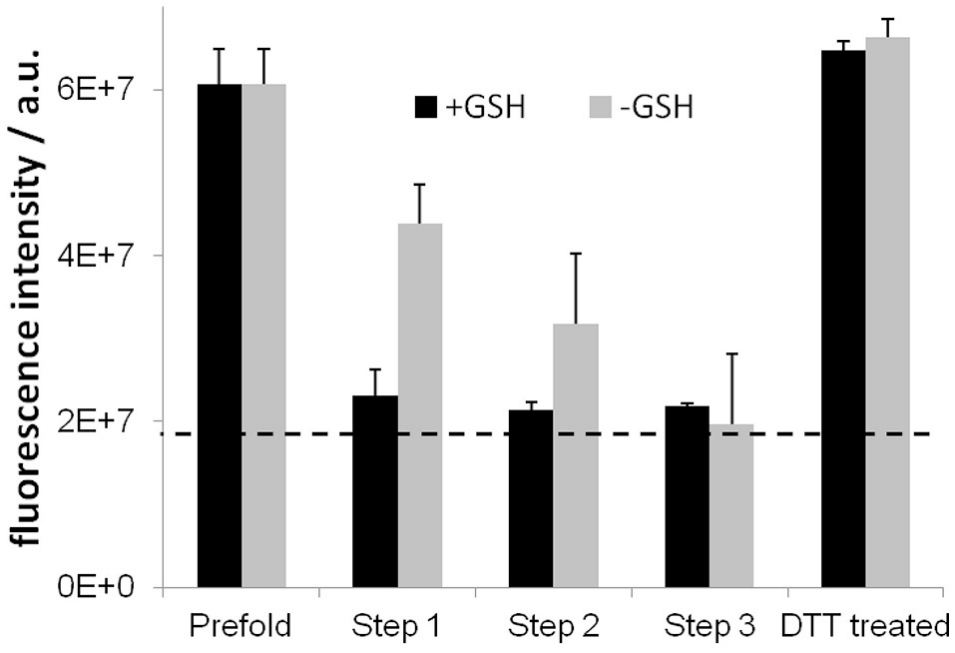
Results of the CPM assay for testing disulfide bridge formation. High fluorescence intensities designate free cysteine residues. The dotted line indicates the background fluorescence intensity. Disulfide bridges are formed during the folding process to completeness after step 3. Glutathione (GSH) accelerates the formation, which is fully reversible shown by reducing the cysteines using DTT.

To assess disulfide bridge formation during the three-step folding process the free cysteines were labeled with CPM and detected in fluorescence measurements (Alexandrov et al., 2008), shown in Figure 3. As expected, in the presence of the glutathione based redox-shuffling system, the two remaining cysteines in the Y_2_R sequence are bridged in step 1 folding dialysis almost completely and remains stable over all steps. Surprisingly, also in the absence of glutathione, the cysteines become connected, although to a lesser extent and after a longer time period.

### Fluorescence-based functionality assays in nanomolar concentration

Functionality of Y_2_R samples at nanomolar concentrations was tested in fluorescence based assays to probe the folding yields and accessibility of both sides of the Y_2_R embedded in bicelles, the ligand binding site as well as the G-protein binding site.

**Figure 4:**
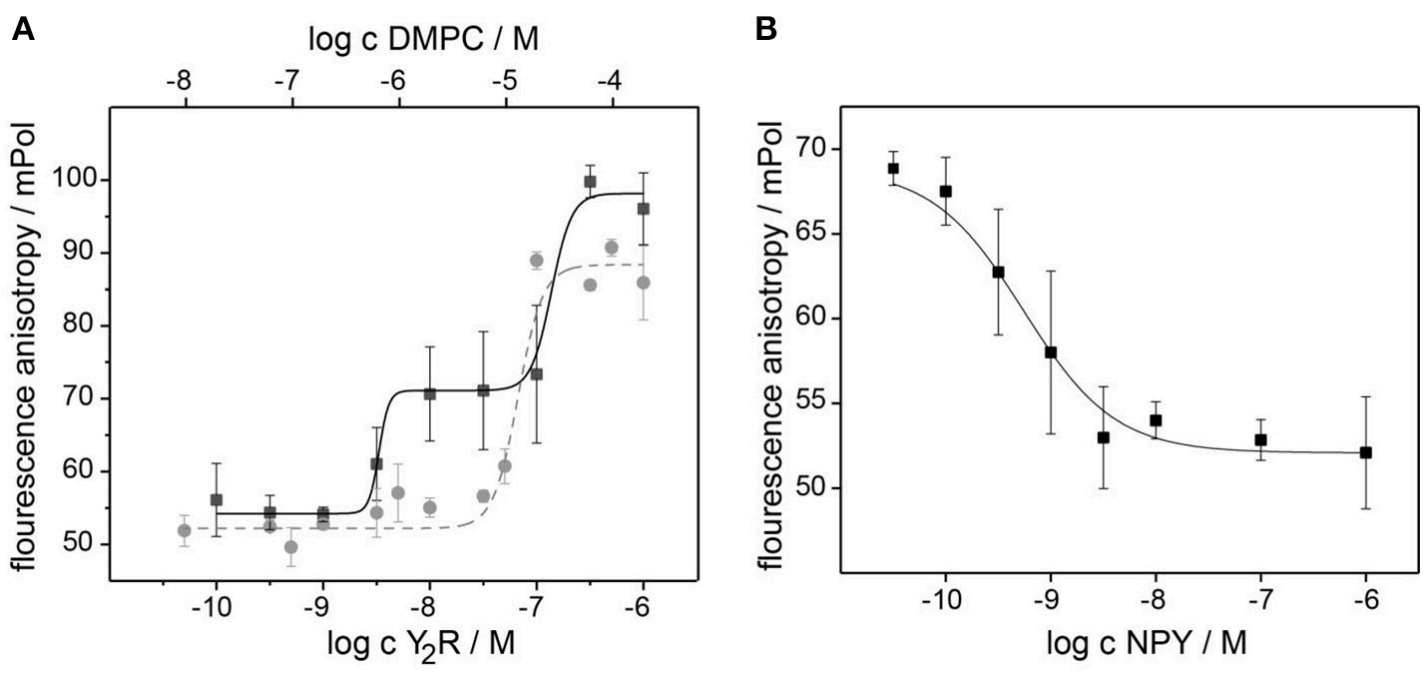
Pharmacological characterization of the Y_2_R preparation at nanomolar concentration using a fluorescence polarization assay with [Dpr^22^-atto520]-NPY. The saturation assay in **(A)** recorded at increasing concentration of bicelle-reconstituted Y_2_R (black) displays two inflection points at 4 and 126 nM. The latter represent the binding of NPY to the membrane, as revealed by the reference measurement with empty bicelles (gray). The specificity of the high affinity Y_2_R binding of 4 nM is confirmed in the competition assay in **(B)** using increasing concentration of unlabeled NPY. The error bars were determined from three independent preparations.

In Figure 4A, saturation curves of NPY binding to Y_2_R and empty bicelles are shown. In the presence of the Y_2_R, two inflection points at values of (4 ± 3) nM and (126 ± 52) nM could be detected from the fit of a two-site binding model to the data points. The higher value displays the binding of NPY to the membrane as shown by the fit of the data points obtained for a pure bicelle preparation in the absence of the Y_2_R. The lower value displays the low nanomolar affinity of the Y_2_R. Binding assays in the presence of 0.1 wt% BSA showed weaker membrane affinities for NPY, but also shifted the affinity to the Y_2_R to higher values (data not shown), implying that pre-binding of NPY to the membrane and hence increasing the effective concentration supports receptor binding (Bader and Zerbe, 2005). Assuming that about 10 DMPC molecules are required to bind one NPY molecule, the affinity of NPY to DMPC membranes is calculated from the inflection point to 2.5 μM. The displacement assay in Figure 4B verified the specificity of the Y_2_R binding, showing an EC50 value similar to the *K*_D_-value in the saturation assay. It is of notice that this assay can only be carried out with a Y_2_R concentration between the two inflection point values determined from the saturation assay, because at lower concentrations no polarization beyond background can be detected and at higher concentrations the membrane binding dominates the measurement, due to higher polarization.

**Figure 5:**
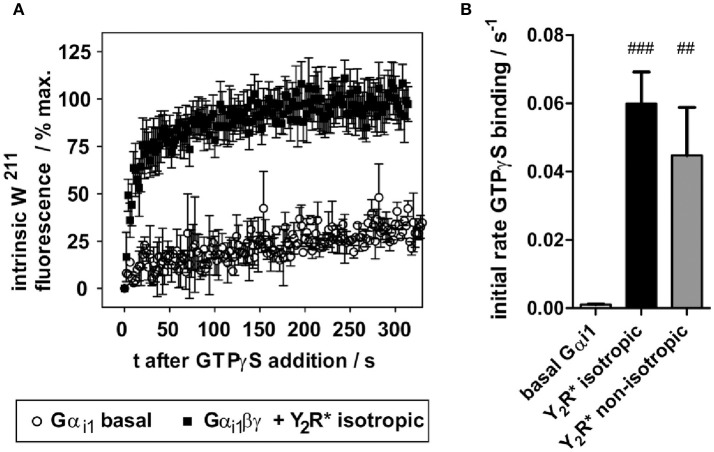
*In vitro* folded Y_2_R variants functionally activate purified G_i_ protein. **(A)** Y_2_R folded into isotropic bicelles and activated with NPY drastically accelerates nucleotide exchange of wild type Gα_i1_. The fluorescence trace is given as mean of seven independent experiments. **(B)** Resulting apparent rates of GTPγS binding of Gα_i1_ (basal) and Y_2_R-catalyzed nucleotide exchange. Statistical significance was determined using one-way ANOVA/Dunnett's post hoc test against basal Gα_i1_ in Graph Pad Prism 5.03. ^###^*p* < 0.001; ^##^*p* < 0.01.

To assess functionality of Y_2_R preparations with respect to G-protein activation, the intrinsic receptor tryptophan fluorescence readout was used, exploiting an activity-dependent increase of W^211^ fluorescence within switch II of Gα_i1_ (Hamm et al., 2009). While W^211^ fluorescence is low in the GDP-bound states, this residue inserts into a hydrophobic pocket upon binding to GTP or GTP analogs, which strongly increases its intrinsic fluorescence. In unbound Gα subunits, nucleotide exchange is very slow, and even essentially absent in G-protein heterotrimers (Gαβγ). Activated GPCRs (R^*^) act as nucleotide exchange factors, when binding Gαβγ-GDP, leading to the high affinity R^*^- Gαβγ “empty” complex, followed by GTP binding and Gα activation (Gα-GTP). In isolated *in vitro* systems, GTPγS can be added to trap Gα in the activated state and to follow GTPγS binding kinetics by tryptophan fluorescence. An exemplary fluorescence trace is shown in Figure 5A. While basal GTPγS binding of Gα_i1_ is very slow (*k* < 0.002 s^−1^), Gα_i1_βγ allowed to interact with the NPY-activated receptor preparations displayed a greatly accelerated nucleotide exchange with apparent GTPγS binding rate of 0.06 ± 0.01 s^−1^. Comparable GTPγS binding rates were also observed in non-isotropic samples (*k* = 0.045 ± 0.014 s^−1^) (Figure 5B).

### NMR experiments to assess receptor function at micromolar concentration

**Figure 6:**
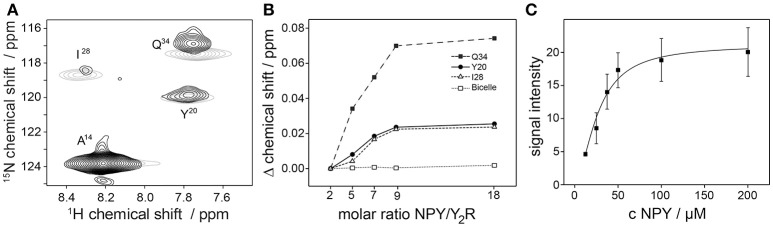
NPY binding tests of the Y_2_R preparation in concentration of 50 μM using solution NMR spectroscopy. In **(A)** a ^1^H-^15^N HSQC spectra of specifically labeled NPY in the presence of Y_2_R with a NPY/Y_2_R ratio of 2 (black) and 18 (gray) are shown. Chemical shift perturbations (CSP) were measured for the labeled NPY positions Y20, I28, Q34 which are involved in Y_2_R binding (Kaiser et al., 2015), but not for A14 which is not interacting with the Y_2_R. As control the same amounts of NPY were titrated to empty bicelles to exclude self-aggregation effects of NPY at high concentration. Concentration dependent binding effects were verified in **(B)** by observing the CSP at increasing concentrations of NPY binding to Y_2_R in isotropic bicelles, and in **(C)** by measuring the signal intensities of bound NPY to Y_2_R in non-isotropic bicelles. All spectra were recorded at 293 K.

Next, we acquired fingerprint NMR spectra of the Y_2_R samples in both preparations, small and large bicelles, at concentrations sufficient for NMR measurements. Ligand binding of the Y_2_R in small bicelles was assessed by recording CSPs of isotopically labeled [^15^N-A^14^,Y^20^,I^28^,Q^34^]NPY in interaction with the receptor (Figure 6). To this end, specifically labeled NPY was titrated to 60 μM of Y_2_R in molar ratios from 2 to 18 and ^1^H-^15^N HSQC spectra were acquired for each sample. The NMR spectrum at the lowest molar ratio compared to the spectrum at the highest ratio is shown in Figure 6A. CSPs were observed for all labeled positions except for A^14^, as it was reported before (Kaiser et al., 2015). Furthermore, weighted chemical shift changes (Δδ = [(Δδ^1^H)^2^ + (0.2 Δδ^15^N)^2^]^1/2^) at different ratios could be measured and were plotted as difference to the chemical shift determined at the lowest ligand to receptor ratio (Figure 6B). Ligand binding on Y_2_R in large bicelles at NMR concentration was tested using a pull-down assay. Varying concentrations of the isotopically labeled NPY were incubated with 40 μM Y_2_R for 2 h, subsequently pelleted and the unbound NPY in the supernatant was removed. The Y_2_R/NPY complex containing pellets were solubilized in SDS to denature the receptor and hence release the NPY. The signal integrals of the NPY, corresponding to the amount, were recorded in ^15^N filtered ^1^H spectra, corrected for intensities measured using empty bicelles, and plotted over the NPY concentration used for incubation (Figure 6C).

Although the determination of binding affinities at micromolar receptor concentration is hardly possible because too many assumptions have to be made, the assays performed here show clear concentration dependent ligand binding effects. Thereby, the presented measurements represent an option to test receptor samples in concentrations required for structural studies.

**Figure 7:**
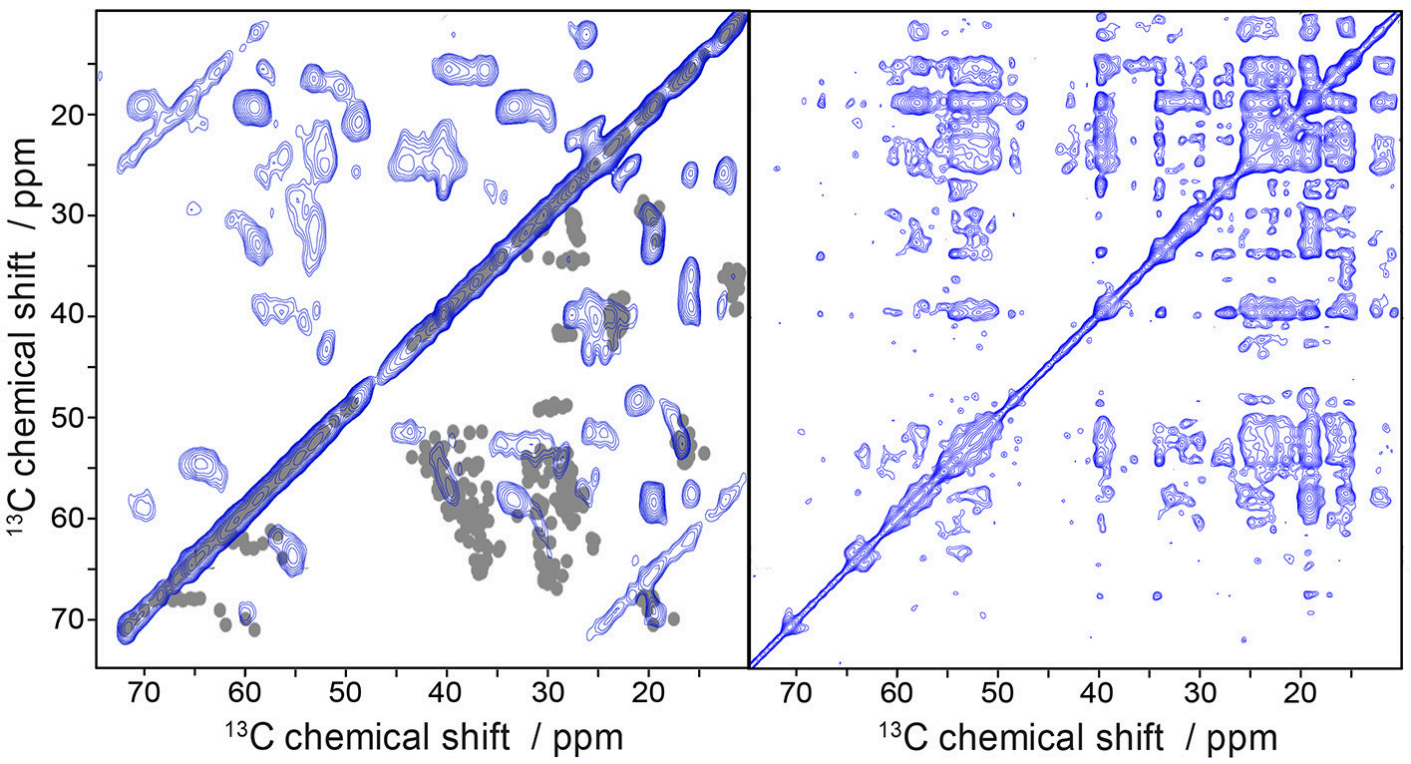
Solid-state MAS NMR spectra of uniformly labeled Y_2_R in non-isotropic bicelles showing ^13^C/^13^C correlation using DARR. The mixing time was varied from 20 ms **(left)** to 500 ms **(right)**. In the bottom right half of the 20 ms DARR spectrum are superimposed one bond correlations cross-signals, simulated from an Y2R homology model. The measurements were performed at a MAS frequency of 7 kHz and a temperature of 5°C.

To finally demonstrate the high efficiency of the folding protocol and the feasibility of the samples for the application to solid-state MAS NMR measurements we recorded ^13^C/^13^C DARR correlation spectra of uniformly ^13^C-labeled Y_2_R (Figure 7) at two mixing times. Already at a mixing time of 20 ms, where polarization can be transferred only between neighboring carbons, a high number of partly resolved signals are visible. Increasing the mixing time to 500 ms allows detecting long range correlations which may indicate tertiary contacts providing valuable constraints for structural studies. Indeed, the number of crosspeaks drastically increases under these conditions.

We predicted one bond correlations (Ca/Cb, Cb/Cg) from a Y2R homology model (Kaiser et al., 2015) in the DARR spectrum at short mixing time using ShiftX2 (Han et al., 2011) and superimposed them with the experimental NMR spectrum (Figure 7). Overall, we found a rather good agreement between experimental and model-based chemical shifts. An interesting exception is that, we find Ala and Leu peaks that indicate beta-sheet like structure. According to the model, the Y2R features one beta-sheet in ECL2 comprising residues 183 to 207. This stretch of amino acids contains two Ala (A184, A202) and two Leu residues (L183, L191), which could produce the beta-sheet like NMR shifts, although ShiftX2 does not predict such chemical shifts. Peak intensity for Ser and Thr residues agrees relatively well with the predicted chemical shifts from the model.

## Discussion

NMR can be a valuable method in structure-based GPCR research, especially when acquiring data on non-engineered receptors in a membrane environment (Wiktor et al., 2013; Ye et al., 2016). Expressing GPCRs in *E. coli* as inclusion bodies and subsequent refolding into membrane environment provides a feasible and successful strategy to obtain the required amounts of isotopically labeled molecules. The first solid-state NMR structural model of a GPCR, the CXCR1 receptor, was determined applying this strategy (Park et al., 2012b). Further, we could recently present a structural model of the peptide NPY bound to the Y_2_R based on NMR restraints from solution and solid-state MAS NMR (Kaiser et al., 2015) and reveal the comprehensive dynamical features of the Y_2_R reconstituted into bicelle environment (Schmidt et al., 2014; Thomas et al., 2015). As a prerequisite for upcoming studies, here, we introduce and discuss an improved folding and preparation protocol, which represents a prerequisite for obtaining structural and dynamical data on the Y_2_R and GPCRs in general.

After inclusion body expression and purification, the GPCRs are generally solubilized in SDS micelles (Baneres et al., 2011). Although the SDS-solubilized receptors are completely non-functional, they already contain most of the native secondary structure including all α-helices, as it has been shown for the BLT1 receptor (Baneres et al., 2003), the μ-opioid receptor (Muller et al., 2008), and the Y_2_R (Schmidt et al., 2009). While the presence of 15 mM anionic detergent SDS suppresses crucial intramolecular contacts for native tertiary structure resulting in non-functional receptor, it prevents aggregation and oligomerization thereby rendering it a good starting point for *in vitro* folding.

During *in vitro* folding of the Y_2_R for NMR measurements, three major steps have to be applied: (*i*) removal of the denaturing SDS without losing receptor molecules by aggregation, (*ii*) formation of the native disulfide bridge between two cysteines in TM3 and ECL2, and (*iii*) high yield reconstitution of the receptor into a stable environment, in which micro- to millimolar protein concentrations can be achieved.

To remove the SDS, its concentration was decreased to 1 mM in step 1 of the folding process. This concentration is below the CMC of SDS, which was determined to 1.9 mM under these conditions (Witte et al., 2013). Further, the Y_2_R concentration was adjusted to a SDS/Y_2_R ratio of 100. Interestingly, this is on the order of the SDS aggregation number, specified with 62–101 molecules per micelle under similar conditions (le Maire et al., 2000). This suggests that the Y_2_R is not kept in a detergent micelle at step 1 of the folding process. What is conceivable instead is that the hydrophobic regions of the molecule are covered by a few SDS molecules, which apparently have a high affinity to the receptor. The hydrophobicity of the receptor seems to have a strong effect on the equilibrium of SDS between the monomeric and the micellar state. Similar effects have been reported on helical domains (Tulumello and Deber, 2009; Alvares et al., 2012). The fact that the Y_2_R is not covered in a large micelle might enable the high yield reconstitution into the phospholipid bilayer. In step 3 of the folding process, the residual SDS was removed below detection limit of ^1^H NMR in the final sample.

A straightforward strategy for an effective formation of the native disulfide bridge between the two cysteines in TM3 and ECL2 and prevention of non-functional bridging between free cysteines is to reduce their number in the sequence to the possible minimum (Li et al., 2008; Wiktor et al., 2013). Following this strategy for the Y_2_R, all cysteines were exchanged to serine or alanine except for the two cysteines involved in the required disulfide bridge. Fortunately, these mutations did not interfere with the functionality of the receptor (Witte et al., 2013). Interestingly, even the mutation of the putative palmitoylation site at the C-terminus did not alter cell surface expression and signaling properties of the Y_2_R (Walther et al., 2012). We achieved the complete disulfide bridge formation in step 1 of the folding process accelerated through the use of glutathione. This is important because at this step the monomeric Y_2_R is still protected by ionic SDS and therefore the formation of intermolecular non-native disulfide bridges is avoided.

We reconstituted the Y_2_R into DMPC/DHPC-c7 bicelle-like structures, which is known to represent a much more stable environment for GPCRs than detergent micelles (Bosse et al., 2011; Witte et al., 2013). Similar to SMA- or MSP-nanodiscs (Casiraghi et al., 2016; Logez et al., 2016; Ravula et al., 2017), both intra- and extracellular sides of the receptors are accessible in bicelles for interacting molecules, whereas in liposomes there is only one. This enables detection of ligand as well as G-protein interaction. Additionally, the size of the bicelles can be adjusted by varying the *q*-value (molar DMPC/DHPC-c7 ratio) from isotropically tumbling bicelles (*q* < 0.25) to large non-isotropic membrane structures with little residual detergent (Son et al., 2012). Therefore, very similar preparations of receptors in bicelles can be used in complementary methods of solution and solid-state NMR (Kaiser et al., 2015). Especially, in our preparation of non-isotropic bicelles, the Y_2_R is densely packed within the membrane. Considering the given lipid/Y_2_R ratio and assuming cross-sectional areas of 60 Å^2^ for DMPC, 2000 Å^2^ for the Y_2_R and 10^7^ Å^2^ for one bicelle, we can estimate that each receptor is surrounded by a lipid annulus comprising 2 to 3 molecular layers.

Using fluorescence polarization assays, strong affinities of NPY to the Y_2_R with an apparent *K*_D_-value of (4 ± 3) nM, but also of NPY to the DMPC membrane of 2.5 μM, assuming ten DMPC molecules bind to one NPY molecule, were calculated. Unfortunately, the determination of the high affinity inflection point is limited by the concentration of the labeled ligand and thus does not provide a true *K*_D_. Due to the limitations in fluorescence detection, the binding assay is conducted far above the expected sub-nanomolar equilibrium binding constant with labeled ligand and receptor present in the same concentration range. Under these conditions, the apparent *K*_D_ is no longer independent of the number of binding sites to be saturated and deviates from true *K*_D_, as the apparent *K*_D_ = true *K*_D_ + $\frac{1}{2}$ [Ligand] (Hulme and Trevethick, 2010). Thus, $\frac{1}{2}$ [Ligand] represents the assay limit assuming a very high affinity *K*_D_. Within experimental error including concentration determination of labeled ligand and receptor, this limit is met for the Y_2_R preparations. Thus, high affinity binding of the receptor can be concluded, with a *K*_D_ that is at least in the low nanomolar range.

The measured fluorescence anisotropy of the atto520 labeled NPY corresponds to the degree of freedom of the fluorescence label. Therefore, in the bound state the label has a lower degree of freedom and hence higher anisotropies are expected for NPY. Surprisingly, higher anisotropies were detected for the membrane bound NPY than the receptor bound NPY. The NPY was labeled on position 22 within the C-terminal α-helix. When bound to the membrane, large parts of the NPY helix are in contact with the phospholipids (Bader and Zerbe, 2005) and the NPY molecule lays flat on the membrane, restricting the NPY diffusion to the two-dimensional surface. In contrast, the structural model of NPY bound to the Y_2_R shows a rather steep pose of NPY with respect to the membrane normal, where only the C-terminal part is interacting with the receptor and the helical parts, including residue 22, sticks out into the solution (Kaiser et al., 2015).

Before collecting data on the structure and dynamics of GPCRs using NMR spectroscopy, it is necessary to prove functionality of the receptor samples in high concentrations as used in NMR spectroscopy. However, a comprehensive pharmacological characterization in terms of affinities or *K*_D_-values is difficult under these concentrations. Here, we present two approaches to confirm a concentration dependent binding response using NMR measuring either CSPs (Kunze et al., 2016) or intensities of specifically labeled NPY bound to Y_2_R. Because of the size and the dynamic features of wild type GPCRs (Latorraca et al., 2017), the NMR spectra are dominated by short *T*_2_ relaxation times, resulting in relatively large line widths and thus low spectral resolution in solution NMR (Wiktor et al., 2013) as well as in solid-state NMR (Schmidt et al., 2014). Using peptides with only a few labeled amino acids reduces the number of signals in each NMR spectrum, thereby providing a simple way to avoid signal overlap and enabling straightforward signal assignment. This approach was used in the binding assays. However, in first solution NMR experiments, the signals of amino acids involved in Y_2_R binding were strongly broadened and signal intensities dropped below detection limit. To still be able to assign these important amino acids in the bound state, we have used a minimum of twofold molar excess of NPY in solution NMR. Therefore, the obtained signals represent an average of the signal intensities from bound and free NPY. In consequence, CSPs presented in Figure 6 also represent an average and would even be higher for the bound NPY assuming fast exchange on the NMR time scale.

Finally, we recorded first ^13^C/^13^C DARR correlation spectra to prove that receptor loading within the bicelles and hence signal intensities are sufficient for solid-state MAS NMR measurements. Both short- and long range proximities can be probed by this approach, provided (nearly) full signal assignment can be achieved. Given the high degree of control over synthesis pathways during *E. coli* protein expression using appropriately labeled precursors, specifically isotopically labeled receptor variants can be produced which should simplify the NMR assignment (Castellani et al., 2002; Loquet et al., 2011, 2013).

In conclusion, we present a robust and efficient protocol for functional reconstitution of the Y_2_R into either isotropic or non-isotropic phospholipid bicelles. The preparations provide the receptor concentrations required for spectroscopic methods, like solution and solid-state NMR or EPR. The protocols can be adapted to other GPCRs as already shown for the GHSR (Schrottke et al., 2017). Further, samples can not only be prepared from GPCRs expressed in *E. coli* as inclusion bodies, but also from all other precipitated GPCRs, such as from cell-free expression produced in the PCF-mode (Rues et al., 2016).

## Author contributions

PS developed folding protocol, supervised experiments, and wrote the manuscript. BB recorded EM images and performed CPM assay. AK performed G protein activation assay and peptide syntheses. KG performed FP assay. HS recorded NMR spectra. BB, HH, JM, AB-S, and DH revised the manuscript.

### Conflict of interest statement

The authors declare that the research was conducted in the absence of any commercial or financial relationships that could be construed as a potential conflict of interest.
